# γ-2 and GSG1L bind with comparable affinities to the tetrameric GluA1 core

**DOI:** 10.1186/s11658-023-00470-9

**Published:** 2023-07-10

**Authors:** Chenlu Yu, Hendrik F. P. Runge, Antara Mukhopadhyay, Gerd Zolles, Maximilian H. Ulbrich

**Affiliations:** 1 Internal Medicine IV, Department of Medicine, University Medical Center, and Faculty of Medicine, University of Freiburg, Freiburg, Germany; 2 BIOSS Centre for Biological Signalling Studies, University of Freiburg, Freiburg, Germany; 3 Institute of Physiology, Faculty of Medicine, University of Freiburg, Freiburg, Germany

**Keywords:** Receptor assembly, Subunit stoichiometry, AMPA receptor regulatory subunits, Single-molecule imaging

## Abstract

**Background:**

The AMPA-type ionotropic glutamate receptor mediates fast excitatory neurotransmission in the brain. A variety of auxiliary subunits regulate its gating properties, assembly, and trafficking, but it is unknown if the binding of these auxiliary subunits to the receptor core is dynamically regulated. Here we investigate the interplay of the two auxiliary subunits γ-2 and GSG1L when binding to the AMPA receptor composed of four GluA1 subunits.

**Methods:**

We use a three-color single-molecule imaging approach in living cells, which allows the direct observation of the receptors and both auxiliary subunits. Colocalization of different colors can be interpreted as interaction of the respective receptor subunits.

**Results:**

Depending on the relative expression levels of γ-2 and GSG1L, the occupancy of binding sites shifts from one auxiliary subunit to the other, supporting the idea that they compete for binding to the receptor. Based on a model where each of the four binding sites at the receptor core can be either occupied by γ-2 or GSG1L, our experiments yield apparent dissociation constants for γ-2 and GSG1L in the range of 2.0–2.5/µm^2^.

**Conclusions:**

The result that both binding affinities are in the same range is a prerequisite for dynamic changes of receptor composition under native conditions.

**Supplementary Information:**

The online version contains supplementary material available at 10.1186/s11658-023-00470-9.

## Background

The α-amino-3-hydroxy-5-methyl-4-isoxazolepropionic acid (AMPA) receptor is pivotal in regulation of synaptic transmission and higher cognitive processes. Its auxiliary subunits have attracted considerable attention in the past 10 years, but their interplay to shape synaptic transmission is far from being understood. Cryo-EM structures of the receptor core, together with the auxiliary subunits γ-2, γ-8, GSG1L, CNIH2, and CNIH3, were published in recent years [1–5]. The structures show that these auxiliary subunits all bind to the same position with respect to the receptor core, between the helices M1 and M4 of two adjacent core subunits, and even share the same topology of the helices. Therefore, they should be expected to compete for binding to the receptor core. Although the side chains that form the contact points between the core subunits and the auxiliary subunits are visible in the structures, it remains impossible to predict the strength of the binding because other factors beyond the interaction energy play a role in the regulation of the binding affinity.

In this study, we investigate binding of the auxiliary subunit GSG1L to the receptor core composed of four GluA1 subunits and its interplay with the auxiliary subunit γ-2. Using single-molecule imaging in *Xenopus laevis* oocytes, we find that up to four fluorescently labeled GSG1L subunits can bind to the receptor core. When unlabeled γ-2 is coexpressed in excess, it can displace GSG1L from the core. Likewise, unlabeled GSG1L can displace fluorescently labeled γ-2 from the core. To determine the relative binding propensities of auxiliary subunits to the core, we simultaneously imaged the core and both auxiliary subunits. For this purpose, we established a three-color single-molecule imaging approach, where γ-2 is labeled with mCherry, GSG1L with mNeonGreen, and GluA1 with a nanobody that binds to a non-fluorescent GFP tag and carries a far-red organic dye. Counting the photobleaching steps of the mNeonGreen-labeled auxiliary subunit bound to the receptor core, while measuring the membrane density of the mCherry-labeled auxiliary subunit, yields a density-dependent displacement curve. We find that the apparent dissociation constants for both γ-2 and GSG1L are in the range of 2.0–2.5/µm^2^. Our observation of direct interplay of AMPA receptor auxiliary subunits in a living cell without the use of concatenated fusion constructs paves the way for a better understanding of the dynamic modulation of AMPA receptor composition.

## Methods

### Constructs for fluorescently tagged membrane proteins and nanobody

For C-terminal fusions to GluA1 (rat, isoform X1, accession number XP_032769863.1), γ-2, or GSG1L, the linker “SRGTSGGSGGSRGSGGSGG” separated the target proteins from C-terminally fused GFP, GFP (Y66L), mCherry, or tdCherry, and the linker “SRGTSGGTG” from mNeonGreen. For an N-terminal fusion to GluA1, GFP was inserted at position 20 after the start codon, i.e., after the signal peptide that gets cleaved; here, a linker sequence “GGSGGRT” was inserted between GFP and the remainder of the GluA1 sequence. All constructs were cloned into pGEMHE, which contains the 5′ and 3′ untranslated regions from *Xenopus* globin [6]. For the nanobody expression vector pET22b-pelB-K4-vhhGFP4-6xHis, a linker containing four lysine residues for dye conjugation and the anti-GFP nanobody (Addgene #35579) were cloned into the pET22b vector (Novagen, EMD Millipore) (full sequence see Additional file 1: Note 3).

### Nanobody expression

For nanobody expression, a 500 mL suspension culture of *E. coli* strain BL21(DE3) transformed with the plasmid pET22b-pelB-K4-vhhGFP4-6xHis was grown to OD600 0.8 at 37 °C, induced with IPTG, and incubated for 16 h at 18 °C. Cells were harvested by centrifugation and lysed by sonication in buffer A (20 mM imidazole, 300 mM NaCl, 50 mM Tris, 10% (w/w) glycerol, pH 8) supplemented with protease inhibitors (cOmplete ULTRA tables Mini, Roche). Following centrifugation at 30,000*g* and 4 °C for 45 min, nanobodies were purified by high-performance liquid chromatography (HPLC) on a His Trap column (5 mL, GE Healthcare). For His-tag purification, the column was equilibrated with buffer A (see above), the sample loaded and washed with 97% buffer A and 3% buffer B [400 mM imidazole, 300 mM NaCl, 50 mM Tris, 10% (w/w) glycerol, pH 8], and eluted in 40% buffer A/60% buffer B. The buffer was exchanged using gel filtration (HiTrap Desalting 5 mL, GE Healthcare). Nanobody size was confirmed by SDS–PAGE.

### Fluorescent nanobody labeling with Alexa Fluor 647

The nanobody was dialyzed into 0.2 M NaHCO_3_ (pH 8.3, mini-dialysis unit MWCO 3.5 kDa, Pierce). Alexa Fluor 647 NHS ester (Invitrogen) dissolved in anhydrous DMSO was added in 10× molecular excess and incubated for 2–4 h at room temperature. Reactions were cleaned up with desalting columns (Zeba spin 0.5 mL 7 k MWCO, Pierce). To estimate the degree of labeling, i.e., the average number of dye molecules per nanobody, the concentrations of protein (using absorbance at 280 nm, A280, molar extinction coefficient ε = 27,055 M^−1^ cm^−1^, weight 27.1 kDa) and AF647 (A650, ε = 239,000 M^−1^ cm^−1^, weight 1.3 kDa) were determined separately; to account for AF647 absorbance at 280 nm, a correction factor of 0.03 × A650 was subtracted.

### *Xenopus* oocyte preparation

**Fig. 1:**
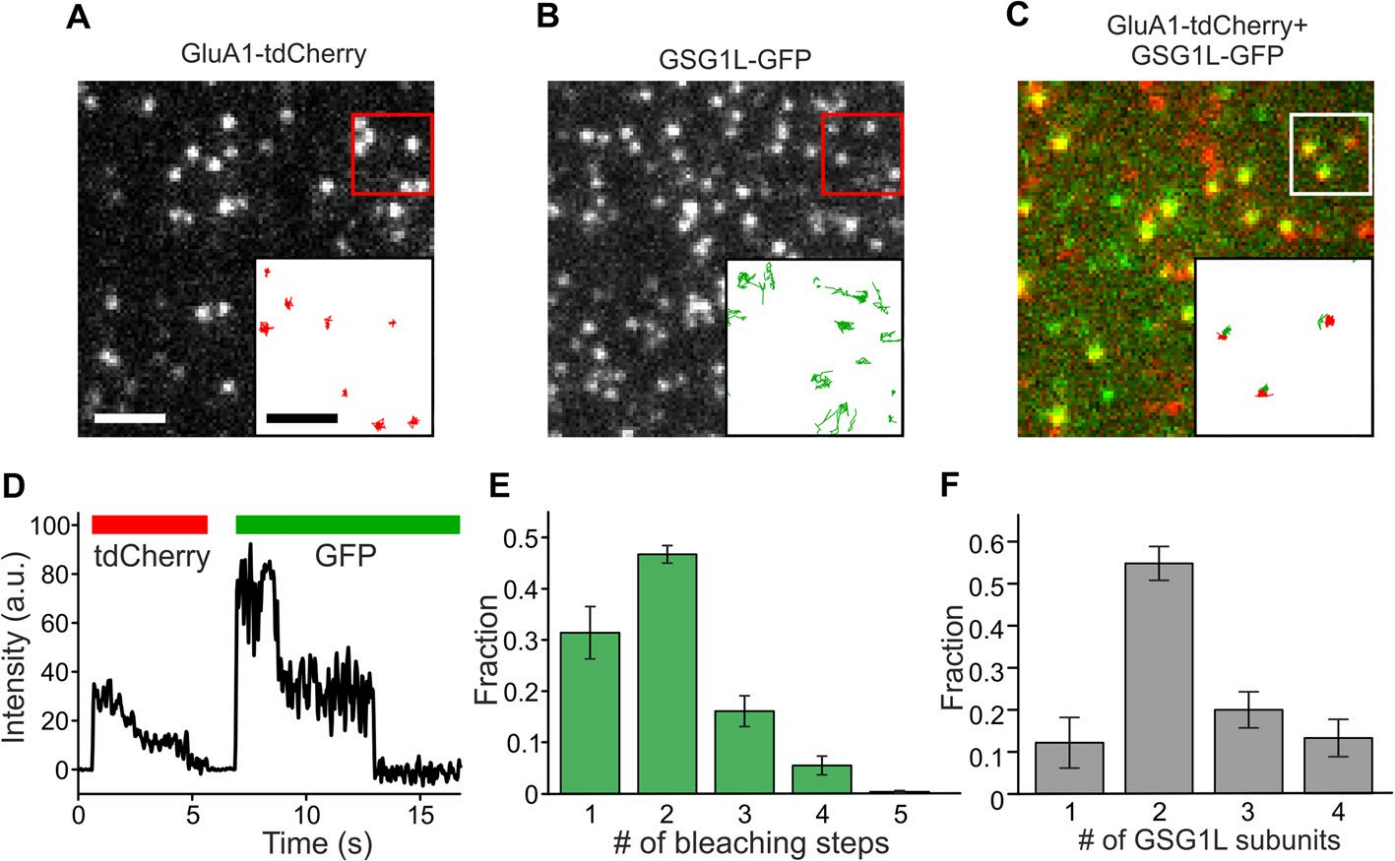
Assembly of GluA1-tdCherry with GSG1L-GFP. **A** On a single-molecule level, GluA1-tdCherry tetramers appear as bright spots in the *Xenopus* oocyte membrane. Tracking their position (inset) suggests that they are immobile. **B** In contrast, GSG1L-GFP spots have a higher mobility. **C** When coexpressed, GluA1-tdCherry (red) and GSG1L-GFP (green) colocalized, and the latter became immobile. **D** The intensity of an individual spot where GluA1-tdCherry and GSG1L-GFP colocalized. Bars above the trace indicate emission in the respective channel. While in the GFP channel, individual photobleaching steps can be identified, the tdCherry emission appears as a decay without individual steps. **E** GSG1L-GFP photobleaching steps in the colocalizing spots. **F** Correction for non-fluorescent GFP suggests that up to 4 GSG1L-GFP can bind to each GluA1-tdCherry core. Scale bars 2 µm and 1 µm (insert)

**Fig. 2:**
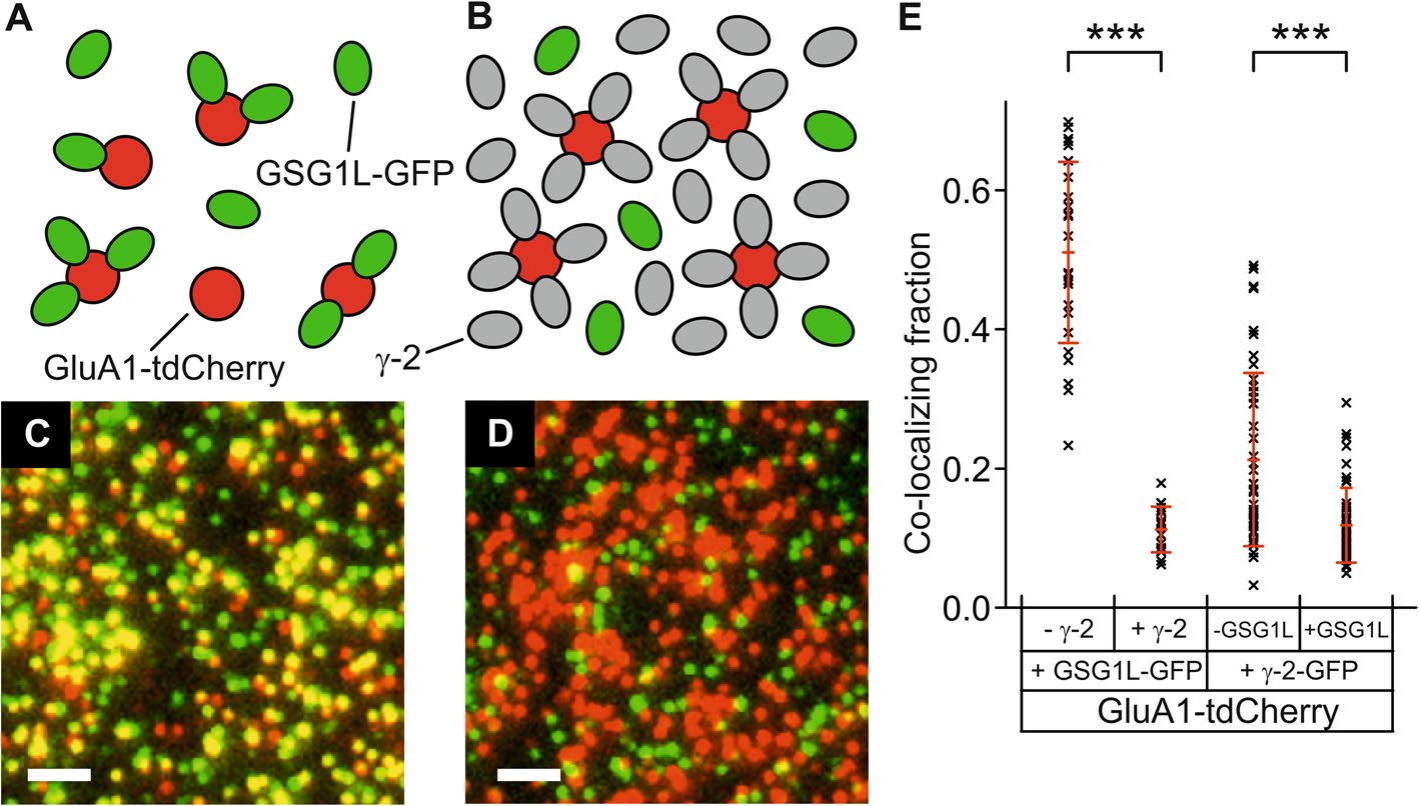
γ-2 displaces GSG1L from binding sites. **A**, **C** Coexpression of GluA1-tdCherry and GSG1L-GFP yields mainly yellow spots, and a few green and red spots. **B**, **D** When unlabeled γ-2 is coexpressed in large excess with GluA1-tdCherry and GSG1L-GFP, it displaces GSG1L-GFP from binding sites, and mainly green and red spots remain. Scale bars 2 µm. The intensities at the spot centers in **C** and **D** are moved to the saturated range to better visualize the green/red overlap, or lack thereof. **E** Colocalization fraction (mean ± s.d.) when GluA1-tdCherry is coexpressed with GSG1L-GFP or γ-2-GFP, with or without unlabeled γ-2 or GSG1L, respectively

**Fig. 3:**
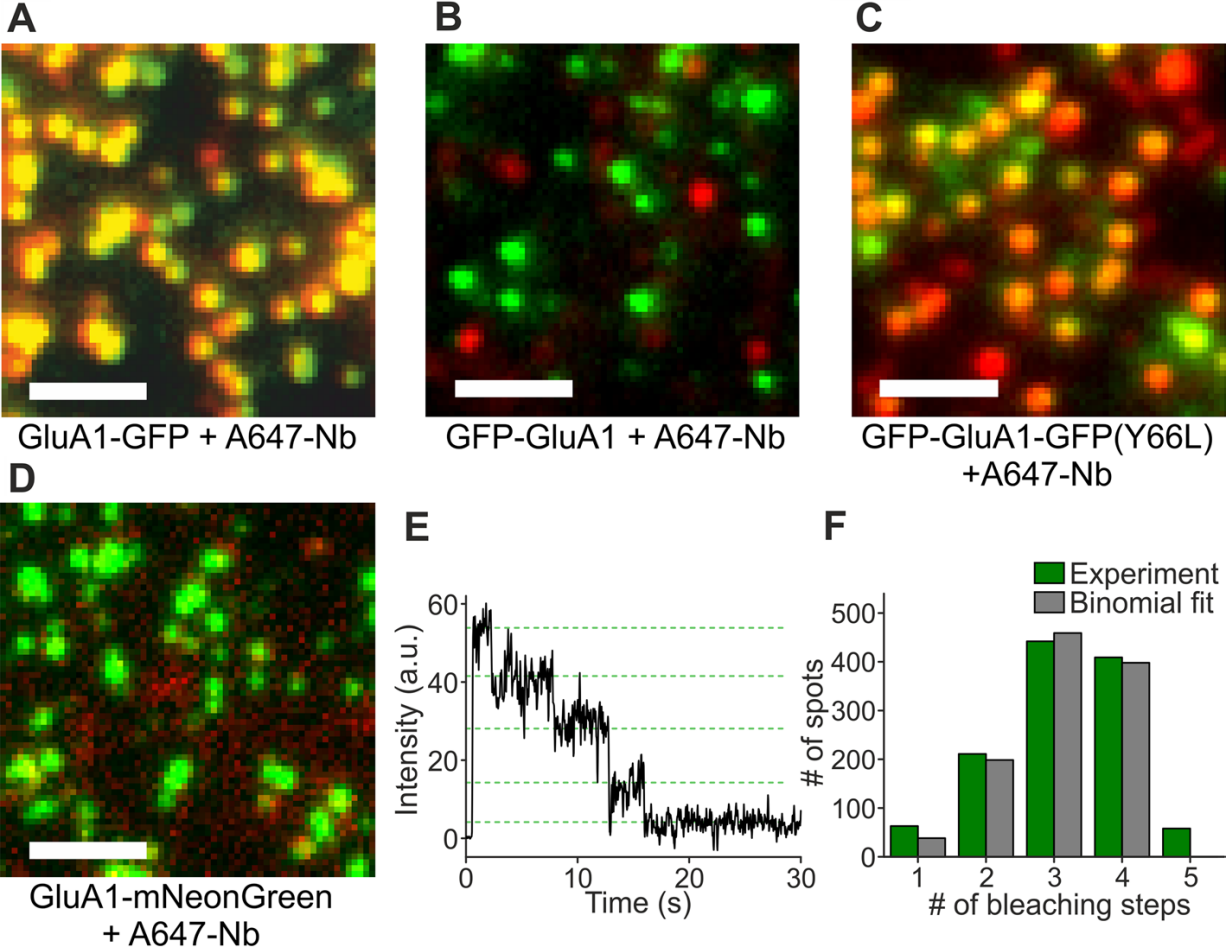
The anti-GFP nanobody binds GFP but not mNeonGreen. **A** A647-Nb injected into oocytes expressing GluA1-GFP shows strong colocalization with GluA1-GFP. **B** A647-Nb does not label the extracellular GFP of GFP-GluA1. Weak red spots in the background are from intracellular A647 molecules. **C** A647-Nb binds to the non-fluorescent GFP(Y66L) of GFP-GluA1-GFP(Y66L). **D** A647-Nb does not label intracellular mNeonGreen of GluA1-mNeonGreen. **E** Photobleaching steps of GluA1-mNeonGreen. **F** A representative distribution of mNeonGreen photobleaching steps with a binomial fit. Scale bars 2 µm

**Fig. 4:**
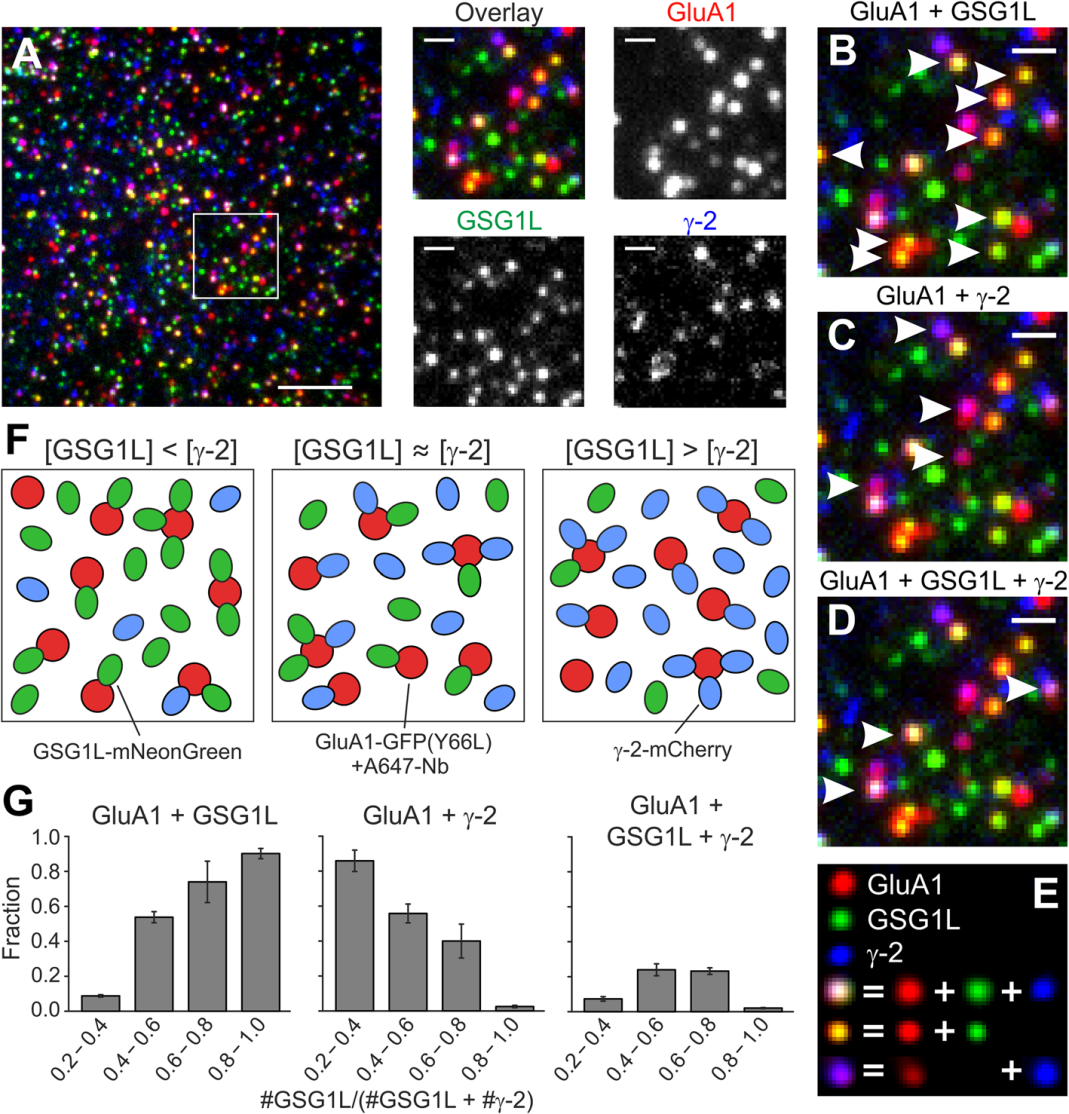
Direct observation of GSG1L and γ-2 competing for binding sites at GluA1. **A** In cells coexpressing GluA1-GFP(Y66L), GSG1L-mNeonGreen (visualized in green), and γ-2-mCherry (blue), and injected with A647-Nb (red), all three colors colocalize in some spots. **B**–**D** Arrowheads mark spots where (labeled) GluA1 and GSG1L (**B**), GluA1 and γ-2 (**C**), or GluA1, GSG1L and γ-2 (**D**) colocalize. **E** Explanation of pseudocoloring in **A**–**D**. **F** Depending on the ratio of GSG1L and γ-2, there is either preferential colocalization of GluA1/GSG1L (left), GluA1/γ-2 (right), or both, including three-color colocalizations (middle). **G** Fractions of colocalizing spots for different GSG1L/(GSG1L + γ-2) spot number ratios as indicated on the *x*-axes. Values for “GluA1 + GSG1L” and “GluA1 + γ-2” include three-color colocalizations (≥ 3 movies for each condition, error bars s.e.m.). Scale bars 5 µm (full view in **A**), 1 µm (magnified views in **A**–**D**)

**Fig. 5:**
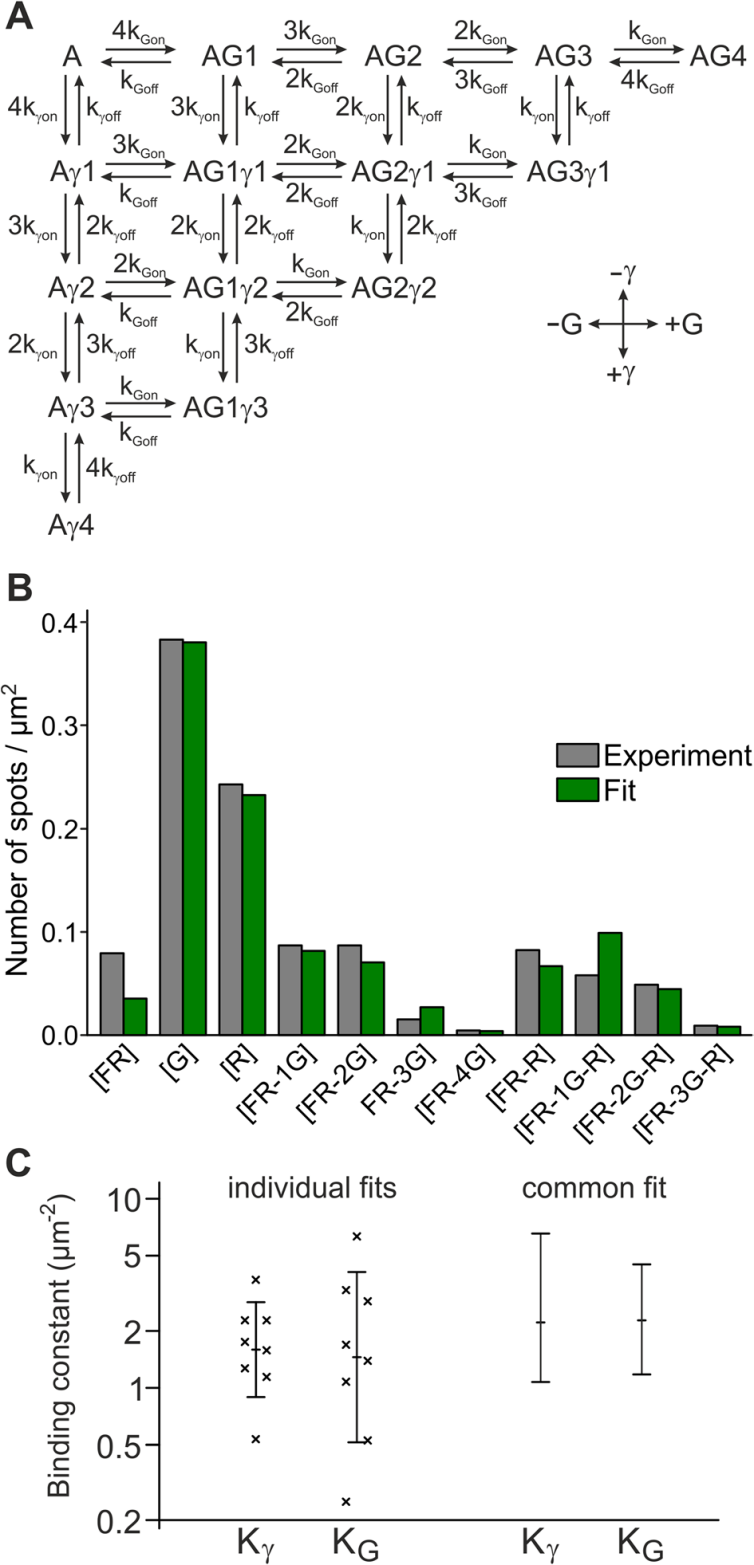
Determination of binding constants K_γ_ and K_G_ for γ-2 and GSG1L to the receptor core. **A** State diagram with transition rates for an AMPA receptor core (A) with different numbers of GSG1L (G) and γ-2 (γ) bound (e.g., AG1γ2 means “core with one GSG1L and two γ-2 bound”). **B** Fit of the state model to the experimental spot densities for an individual representative experiment. FR, G, and R refer to the presence of far-red fluorescence [A647-Nb bound to GluA1-GFP(Y66L)], the number of green bleaching steps (GSG1L-mNeonGreen), and the presence of red fluorescence (γ-2-mCherry), respectively. **C** Mean values from eight experiments and results of a common fit (maximum likelihood values and 68% confidence intervals) for the binding constants K_γ_ and K_G_

Stage V–VI *Xenopus laevis* oocytes were surgically extracted as previously described [7]. Capped RNA synthesized by in vitro transcription (mMESSAGE mMACHINE T7 Kit, AM1344, ThermoFisher) was injected 18–24 h before experiments. Amounts of injected RNA (in 50 nl volume) were: 5 ng GluA1-tdCherry + 25–100 pg GSG1L-mEGFP (Fig. 1), 5 ng GluA1-tdCherry + 12.5–25 pg GSG1L/γ-2-mEGFP + 0.5–1 ng GSG1L/γ-2 (Fig. 2), 5 ng GluA1-mNeonGreen (Fig. 3), and 2.5–5 ng GluA1-mEGFP(Y66L) + 2.5–25 pg GSG1L-mNeonGreen + 2.5–50 pg γ-2-mCherry (Figs. 4, 5). When used, 0.1 pmol A647-Nb was injected 2 h before the experiment, leading to a final intracellular concentration of ~ 100 nM when assuming an oocyte volume of 1 µl.

### Movie acquisition

In principle, experiments and analysis were done as previously described [7–9]. After removing the vitelline membrane with two Dumont #5 forceps, the oocytes were placed on a high refractive index coverslip (*n* = 1.78) and movies of 1000–1500 frames were recorded at 33 Hz using a back-illuminated EMCCD camera (Andor iXon897) connected to an inverted microscope (Olympus IX71) equipped with an 100x/NA1.65 or NA1.70 objective (Olympus). A 25.6 µm × 25.6 µm area of the sample (effective pixel size 100 nm, 1.6 × tube lens magnification applied) was illuminated in total internal reflection configuration with a 637 nm laser to excite Alexa Fluor 647, followed by a 561 nm laser to excite mCherry and a 488 nm laser for GFP or mNeonGreen at power densities of 90–150 W/cm^2^. Emission was recorded through a multiband dichroic (Semrock Di01-R405/488/561/635) and one of three additional single-bandpass filters that were switched between the sections of the recording (red: Chroma ET585/65 m; far-red: Semrock FF01-629/53; green: Semrock FF01-525/50). Crosstalk between channels was not observed.

### Data analysis

We selected recordings from oocytes expressing receptors at densities below 500 spots per frame (for Fig. 2: below 625 spots) to obtain a low probability that two receptors would lie within one diffraction-limited spot. Only immobile receptors were evaluated; the mobile fraction was small, usually < 20%. We extracted the emission intensities over time from the GFP or mNeonGreen channel and manually counted discrete bleaching steps. To assess the overlap of green, red, and far-red, the first five images of each channel were superimposed and spots were defined as colocalized when they were closer than two pixels (200 nm). The number of GSG1L subunits bound to the GluA1 core (Fig. 1F) was calculated for individual experiments from the distribution of bleaching steps according to ref. [8], assuming a probability of 0.8 of GFP to be fluorescent. For the common fit (Fig. 5), confidence intervals were determined according to ref. [10].

### Electrophysiology

Electrophysiological recordings from outside-out patches excised from *Xenopus* oocytes were performed at room temperature (22–24 °C), as described previously [11]. cRNAs of GluA1 (flip), GluA2 (flip), and either GSG1L or GSG1L-GFP were injected at a ratio of 1:1:4. Currents were recorded with an EPC10 amplifier, low-pass filtered at 3 kHz, and sampled at 1–10 kHz. Pipettes made from thick-walled borosilicate glass had resistances of 0.4–1.2 MOhm when filled with intracellular solution (116 mM of KCl, 1.1 mM of MgCl_2_, 10 mM of HEPES, and 2 mM of EGTA, with pH adjusted to 7.2). Extracellular solution (ES) applied to outside-out patches was composed as follows: 116 mM of KCl, 1.1 mM of MgCl_2_, 10 mM of HEPES, and 2 mM of EGTA (pH 7.2). Rapid application/removal of glutamate (10 mM dissolved in ES) was performed with a piezo-controlled fast application system with a double-barrel application pipette that enables solution exchanges within less than 100 µs (20%–80%), measured by switching the open tip of the giant patch pipettes between normal and 10 × diluted ES. Currents were recorded at −70 mV holding potential. Desensitization and recovery from desensitization were characterized by time constants derived from mono-exponential fits to the decay phase or recovery of the respective currents.

## Results

### Auxiliary subunits γ-2 and GSG1L associate with the GluA1 core

Single-molecule imaging and counting of GFP photobleaching steps demonstrated previously that the transmembrane AMPAR regulatory proteins (TARPs) γ-2, γ-3, γ-4, and γ-8 associate with the tetrameric GluA1 core with a stability in the range of at least several seconds, probably longer, and up to four γ-2 or γ-3 can bind to one receptor core [8]. In contrast, a maximum of two γ-4 or γ-8 were found to bind to the receptor core, suggesting that the two-fold symmetry of the receptor leads to two different types of binding sites, and the maximum occupancy of the binding sites depends on the auxiliary subunit type.

We therefore set out to determine the number of binding sites at the GluA1 core that can be occupied by the auxiliary subunit GSG1L. In the subunit counting approach, the protein of interest (in this case GSG1L) is labeled with a (in this case) GFP fusion tag. During imaging, all GFP tags in a protein complex are so close they appear as a single fluorescent spot, but they photobleach sequentially, leading to a stepwise decrease of fluorescence intensity when viewed on a single-molecule level. The number of bleaching steps before complete loss of fluorescence equals the initial number of intact GFP tags [7]. Experiments with reference proteins of known stoichiometry yield a fraction of non-functional GFP tags of around 20% [7]. Using this value, the number of GluA1 tetramers with 0 to 4 GSG1L-occupied binding sites can be estimated from the distribution of observed bleaching steps.

We generated C-terminal fusion constructs of GSG1L with GFP (GSG1L-GFP) and GluA1 with a tandem dimeric mCherry (tdCherry) for increased visibility compared to a single mCherry. Because the effect of GSG1L-GFP on glutamate-evoked currents of GluA1 was similar to the effect of untagged GSG1L, we assumed that the GFP tag had no effect on the function or assembly (Additional file 1: Fig. S1).

First, we expressed GluA1-tdCherry alone in *Xenopus* oocytes and recorded movies of the plasma membrane using total internal reflection fluorescence (TIRF) microscopy. GluA1-tdCherry was visible as immobile red fluorescent spots that photobleached (Fig. 1A). As observed previously, the number of photons emitted by mCherry is too small, and the fluctuations in its emission intensity are too large to see clearly separated photobleaching steps; but in many spots, we observed that the intensity assumed several distinct levels or appeared similar to an exponential decay during photobleaching, suggesting that these spots contained multiple GluA1-tdCherry subunits. Next, we expressed GSG1L-GFP alone and recorded movies (Fig. 1B). In contrast to GluA1-tdCherry, GSG1L-GFP was mobile, and the spots could not be tracked sufficiently long to count bleaching steps, most likely due to the microvilli structure of the *Xenopus* oocyte plasma membrane that leads diffusing molecules out of the focus and the evanescent field of the TIRF illumination [12].

To assess whether GSG1L-GFP interacts with GluA1-tdCherry, we expressed both subunits together, and imaged them sequentially (Fig. 1C). While the GluA1-tdCherry again formed immobile spots that looked the same as when expressed alone, many green spots from the GSG1L-GFP became immobile at the positions of GluA1-tdCherry spots, suggesting that they interact directly. Counting GFP photobleaching steps from spots where GSG1L-GFP colocalized with GluA1-tdCherry yielded up to 4 steps [*n* = 10 movies; 1 step: 31 ± 5% standard error of the mean (s.e.m.); 2 steps: 47 ± 2%; 3 steps: 16 ± 3%; 4 steps: 5.5 ± 1.8%; 5 steps: 0.3 ± 0.2%] (Fig. 1D, E). Correction for the fraction of ~ 20% of non-fluorescent GFP yielded 12 ± 6% spots with one, 55 ± 4% with two, 20 ± 4% with three, and 13 ± 4% with four GSG1L-GFP subunits bound to the GluA1-tdCherry core (Fig. 1F). This supports the view that the GluA1 core provides four binding sites for GSG1L.

In conclusion, our results suggest that up to four GSG1L subunits can bind to the GluA1 core, similar to what was observed previously for γ-2 [8]. Because γ-2 and GSG1L assume the same position when bound to the core (as seen in the structures), they should compete for binding sites [1, 2]. As a side note, we want to point out that the number of GSG1L binding to the tetrameric GluA1 core does not seem to be limited to two, as it has been observed previously by cryo-EM for the GluA2 tetramer [2].

### γ-2 can displace GSG1L from binding sites at the GluA1 core

To assess the anticipated competition between γ-2 and GSG1L for binding sites at the GluA1 core, coexpression of all three subunit types is required. A straightforward strategy is to displace one type of auxiliary subunit from the core by expression of the other type, and observe the resulting decrease in colocalization (Fig. 2A, B). To this end, we coexpressed GluA1-tdCherry and GSG1L-GFP either with or without unlabeled γ-2. To achieve maximal displacement of GSG1L-GFP from the core, we injected the RNA for unlabeled γ-2 at a concentration 20 × higher than in previous experiments. As a measure of colocalization, we counted which fraction of red spots (originating from GluA1-tdCherry) overlapped with green spots (from GSG1L-GFP) within 200 nm. As anticipated, we observed that the large overlap of 51.1 ± 2.6% (*n* = 26 movies; s.e.m.) of green and red without coexpression of unlabeled γ-2 decreased to 11.2 ± 0.8% (*n* = 19) when γ-2 was coexpressed, suggesting that a large fraction of GSG1L-GFP was indeed displaced from the GluA1-tdCherry core (Fig. 2C–E). Conversely, when GluA1-tdCherry and γ-2-GFP were coexpressed, the overlap of green and red decreased upon coexpression of unlabeled GSG1L (from 21.2 ± 1.9%, *n* = 45 to 11.9 ± 0.8%, *n* = 49) (Fig. 2E). The effect was smaller than in the previous experiment, mainly because the binding to the receptor core was less pronounced for γ-2-GFP (when expressed alone) than for GSG1L-GFP (when expressed alone). The reduction of green/red overlap in both cases supports the view that GSG1L and γ-2 are able to displace each other from the binding sites at the GluA1 core.

For a quantitative assessment of the competition, the membrane density of the second unlabeled subunit must be known. However, it is difficult to infer plasma membrane densities of unlabeled subunits from the amount of injected RNA because they are not visible, and in general, expression levels vary between individual oocytes and between different proteins. In addition, the expression levels of tagged and untagged versions of the same protein differ. Thus, we set out to image with three different colors, one for the GluA1 core, one for γ-2, and one for GSG1L.

### Three-color single-molecule imaging with mNeonGreen and labeled anti-GFP nanobodies

For three-color single-molecule imaging, we used the anti-GFP nanobody, which we labeled with the far-red organic dye Alexa Fluor 647 (A647) (Additional file 1: Note 1) [13–15]. After purification, we injected the labeled nanobody (A647-Nb) at a final concentration of 100 nM into the oocytes expressing GluA1-GFP (where the GFP is at the intracellular C-terminus). While the RNA was injected one day before, A647-Nb was injected only 2 h before the experiment. We found that nearly all green spots also displayed far-red fluorescence, suggesting that for most of the GluA1-GFP tetramers, at least one of the four GFP tags interacted with A647-Nb (Fig. 3A). In contrast, when GFP was fused at the extracellular N-terminus of GluA1 (GFP-GluA1), we did not observe significant overlap of green and far-red; in addition, the far-red signal was mainly diffuse and only few dim spots were visible, suggesting that the intracellular A647-Nb did not bind to the extracellular GFP tag (Fig. 3B). Taken together, we can use the anti-GFP nanobody to efficiently and specifically label intracellular GFP tags with a far-red dye.

If we want to use the association of A647-Nb with GFP to tag one target protein with the far-red label, we cannot use GFP at the same time for labeling a different target protein. Therefore, we need a new green tag that is different from GFP, and render the original GFP tag non-fluorescent. To find a non-fluorescent GFP variant, we tested several mutations of the tyrosine at position 66, which is critical for formation of the chromophore, to other amino acids. Of several mutants that did not show fluorescence (data not shown), we chose the variant Y66L. When A647-Nb was injected into cells expressing GluA1-GFP(Y66L), we did not see green fluorescence, but observed bright and immobile far-red spots, similar to our previous experiment where we expressed GluA1-GFP and injected A647-Nb, suggesting that A647-Nb still binds to the GFP mutant (not shown). To confirm that the GFP is present and able to bind A647-Nb, we fused an additional GFP (without the Y66L mutation) to the extracellular N-terminus of GluA1, yielding GFP-GluA1-GFP(Y66L); the N-terminal GFP should not be accessible to the nanobody, as suggested by the previous experiment with GFP-GluA1. Upon expression of GFP-GluA1-GFP(Y66L) and injection of A647-Nb, we observed green fluorescence that colocalized with the far-red signal, suggesting that the non-fluorescent GFP(Y66L) at the AMPA receptor’s intracellular C-terminus is folding properly despite the mutation, and is specifically associating with A647-Nb (Fig. 3C).

As a replacement for GFP, we searched for a green fluorescent protein that is monomeric, bright and photostable, and, to prevent it from binding the nanobody, not derived from the jellyfish *Aequorea victoria*. The seemingly best candidate we found was mNeonGreen, which is derived from *Branchiostoma lanceolatum* [16]. To assess what fraction of mNeonGreen tags is visible under our conditions and whether mNeonGreen is suitable for counting photobleaching steps, we fused it to the C-terminus of GluA1 (GluA1-mNeonGreen) and imaged it in *Xenopus* oocytes. We found that GluA1-mNeonGreen forms bright green fluorescent spots. When A647-Nb was injected into cells expressing GluA1-mNeonGreen, no colocalization of green and far-red was observed, confirming that A647-Nb does not bind to the mNeonGreen tag (Fig. 3D). Furthermore, GluA1-mNeonGreen displayed well discernible bleaching steps and counting of photobleaching steps yielded a fluorescent fraction of 77.2 ± 1.7% (*n* = 5 movies, s.e.m.), similar to GFP (Fig. 3E, F) [7]. Therefore, it is well suited for counting photobleaching steps in single-molecule imaging experiments, and is a good alternative to GFP in our three-color experiments.

### Direct visualization of γ-2 and GSG1L competition

With mNeonGreen, mCherry, and the A647-Nb bound to GFP(Y66L), we now have three fusion tags in different wavelength ranges that we can use for three-color single-molecule imaging of the γ-2/GSG1L competition. The labeling efficiency of A647-Nb bound to GFP(Y66L) might be variable due to the missing control over the effective A647-Nb concentration at the imaged patch of the oocyte. Therefore, we used the GFP(Y66L)/A647-Nb labeling for GluA1, where 4 GFP(Y66L) tags are present, and mCherry and mNeonGreen for labeling γ-2 and GSG1L, respectively. We injected oocytes expressing all three constructs with A647-Nb and recorded movies with consecutive illumination in the three wavelength ranges (Fig. 4A). We observed immobile far-red spots from the A647-Nb bound to GluA1-GFP(Y66L), green spots from GSG1L-mNeonGreen and red spots from γ-2-mCherry; in addition, many spots showed colocalization of two or all three colors (Fig. 4B–E). To observe the anticipated shift from GluA1/GSG1L over GluA1/GSG1L/γ-2 to the GluA1/γ-2 assembly, we varied the amounts of injected RNA for the GSG1L and γ-2 constructs (Fig. 4F). We binned the resulting spot numbers by the fraction of green spots in the total of green and red spots [#GSG1L-mNeonGreen / (#GSG1L-mNeonGreen + #γ-2-mCherry)], in the following referred to as the “GSG1L fraction.” We find that the fraction of spots where GSG1L colocalized with GluA1 increased with an increasing GSG1L fraction, while the fraction of spots where γ-2 colocalized with GluA1 decreased with an increasing GSG1L fraction (Fig. 4G). The fraction of spots where both GSG1L and γ-2 colocalized with GluA1 was small for low and high GSG1L fractions, but increased to about 0.25 for intermediate levels of the GSG1L fraction.

This shift from γ-2/GluA1 to GSG1L/GluA1 colocalization with an intermediate peak of γ-2/GSG1L/GluA1 colocalization with an increasing GSG1L fraction is characteristic of the competition for binding sites. In the next step, we set out to quantify the apparent two-dimensional binding constants of γ-2 and GSG1L to GluA1 at the plasma membrane.

### Relative binding constants of γ-2 and GSG1L to the GluA1 core

To model the binding of γ-2 and GSG1L to GluA1, we assume that the tetrameric GluA1 core has four binding sites that either γ-2 or GSG1L can bind to and that the binding affinity can differ between γ-2 and GSG1L, but is the same for all four sites. The state diagram can be established using on- and off-binding rates ${k}_{\gamma on}$, ${k}_{\gamma off}$ for γ-2 and ${k}_{Gon}$, ${k}_{Goff}$ for GSG1L, with the prefactors according to the number of open or occupied sites (Fig. 5A). When the system is in equilibrium and with binding constants ${K}_{\gamma }={k}_{\gamma on}/{k}_{\gamma off}$ and ${K}_{G}={k}_{Gon}/{k}_{Goff}$, the occupancies of the states can be calculated (Additional file 1: Note 2). For obtaining the observable states, it is important to consider that (i) the probability of mNeonGreen and mCherry to be fluorescent is less than 100%, probably because of incomplete maturation or misfolding, and (ii) mCherry bleaching steps cannot be counted accurately. To account for the non-fluorescent fractions of the fluorescent proteins (i), we defined the probabilities $p$ for mNeonGreen to be fluorescent and $q$ for mCherry to be fluorescent. To account for the inability to count mCherry bleaching steps (ii), all observations containing mCherry fluorescence were pooled. Thus, expressions can be derived that describe the densities of the 11 theoretically possible observations [FR], [G], [R], [FR-1G], [FR-2G], [FR-3G], [FR-4G], [FR-R], [FR-1G-R], [FR-2G-R], and [FR-3G-R]. Here, FR stands for the observation of far-red fluorescence from the nanobody-labeled GluA1-GFP(Y66L) core, 1G…4G for the observation of 1–4 green photobleaching steps from GSG1L-mNeonGreen, and R for the observation of red fluorescence from γ-2-mCherry in one of the spots (Additional file 1: Note 2).

To fit the binding constants ${K}_{\gamma }$ and ${K}_{G}$ to the observed data, we counted the spot numbers for all possible observations in seven individual experiments (Fig. 5B). Division by the area of the imaged region yielded the observed values for the densities [FR], [G], [R], [FR-1G], [FR-2G], [FR-3G], [FR-4G], [FR-R], [FR-1G-R], [FR-2G-R], and [FR-3G-R]. In the model described above, the free parameters are the densities of unbound GluA1 cores [A], free GSG1L [g], and free γ-2 [γ], the binding constants ${K}_{\gamma }$ and ${K}_{G}$, and the probabilities $p$ and $q$ for mNeonGreen and mCherry to be fluorescent. It is important to note that the densities [A], [g], and [γ] are not equal to [FR], [G], and [R], because there are (due to incomplete maturation) invisible free GSG1L-mNeonGreen and γ-2-mCherry molecules that contribute to [g] and [γ], but not to [G] and [R], and GluA1-GFP(Y66L) cores that have only invisible GSG1L-mNeonGreen or γ-2-mCherry bound and therefore contribute to [FR] but not to [A].

The most likely values for ${K}_{\gamma }$ and ${K}_{G}$ were fitted for the individual experiments, where $p=0.77$ (based on our measurement above) and $q=0.65$ (based on our own estimates and [17]) were kept constant, and [A], [g], and [γ] were treated as free parameters. The best fit matched the observed data well, and the resulting values for ${K}_{\gamma }$ and ${K}_{G}$ ranged from 0.54/µm^2^ to 3.7/µm^2^ [mean ± standard deviation (s.d.): 1.8 ± 1.0/µm^2^] and from 0.25/µm^2^ to 6.3/µm^2^ (2.2 ± 2.0/µm^2^), respectively (Fig. 5B, C). We also performed a global fit, where ${K}_{\gamma }$ and ${K}_{G}$ assumed a common value for all experiments, but [A], [g], and [γ] were allowed to vary between the different experiments. We obtained ${K}_{\gamma }$ = 2.2/µm^2^ (68% CI: 1.1–6.6/µm^2^) and ${K}_{G}$ = 2.3/µm^2^ (68% CI: 1.2–4.5/µm^2^) (Fig. 5C). Therefore, the binding strengths of γ-2 and GSG1L to the GluA1 core are not significantly different from each other with apparent dissociation constants in the range of 2.0–2.5/µm^2^. It should be noted that the *Xenopus* oocyte plasma membrane is known to be coiled into a dense mesh of microvilli, effectively increasing the surface by a factor of about 4 [12, 18]. Accordingly, the dissociation constants in a flat membrane would be reduced by that factor.

## Discussion

In this work, we used a single-molecule imaging approach to investigate how AMPA receptor auxiliary subunits compete for binding sites at the receptor core. As exemplary subunits, we used the auxiliary subunits γ-2 and GSG1L and the core subunit GluA1. The direct observation of the competition was facilitated by three-color single-molecule imaging in *Xenopus laevis* oocytes. In addition to the green FP mNeonGreen and the orange-red FP mCherry, we used an anti-GFP nanobody for labeling in the far-red range, which bound to a non-fluorescent GFP(Y66L) fusion tag after injection into the cells. Eventually, we fitted experimentally obtained counts for mNeonGreen photobleaching steps and for colocalizing green, orange-red, and far-red spots with a model for the occupancy of the receptor’s binding sites, using the dissociation constants of γ-2 and GSG1L as fit parameters. Our main findings are that the two subunits can displace each other from the GluA1 receptor core according to the law of mass action, and that the apparent dissociation constants of γ-2 and GSG1L are both in the same range of 2.0–2.5 receptors/µm^2^.

Limitations of our study arise from the fact that within our imaging time of < 60 s, we did not observe a sizeable amount of events where GSG1L or γ-2 subunits dissociated from the receptor core, which means that the interactions are stable within that time frame. On the one hand, this allowed us to reliably count the subunits and determine the colocalization; on the other hand, it prevents us from making conclusions about if or when a new equilibrium state would be reached after a change of one of the subunit densities. In the case of a very stable interaction, competition would occur only at an initial stage, when not all binding sites are occupied yet (possibly already in the endoplasmic reticulum).

It is possible that the stability of the interaction is regulated by additional factors. An early study on TARP/AMPA receptor interactions suggested that γ-2, γ-3, and γ-8 dissociate from the core after agonist binding [19]. Considering the different functional properties of TARPs and GSG1L, this could enable the cell to regulate synaptic properties. GSG1L has no PSD-95 binding domains like TARPs and enhances AMPAR endocytosis, while TARPs enhance surface transport [20, 21]. Therefore, replacement of TARPs by GSG1L under specific cellular conditions could provide a mechanism for removing AMPA receptors from synapses.

## Conclusions

Although a variety of AMPA receptor structures in complex with auxiliary subunits has been determined recently, they draw only a static picture of the assembly. In this study, we analyzed for the first time the interplay of two different AMPA receptor auxiliary subunits in live cells. The result that the binding affinities of γ-2 and GSG1L are in the same range is a prerequisite for dynamic changes of receptor composition under native conditions. Therefore, our work provides important insight into the question of dynamic modulation of the composition of existing receptors.

### Supplementary Information

**Additional file 1.** **Note 1**. Rationale for choosing intracellularly injected nanobodies for labeling. **Note 2**. Model for binding of γ-2 and GSG1L to GluA1. **Note 3**. Full nanobody sequence. **Figure S1**. GFP-labeled GSG1L is functional.

## Data Availability

All data generated or analyzed during this study are included in this published article or are available from the corresponding author upon request.

## Abbreviations

AMPA: α-Amino-3-hydroxy-5-methyl-4-isoxazolepropionic acid
TARPs: Transmembrane AMPAR regulatory proteins
TIRF: Total internal reflection fluorescence
A647: Alexa Fluor 647
A647-Nb: Alexa Fluor 647-labeled anti-GFP nanobody
HPLC: High-performance liquid chromatography
SDS-PAGE: Sodium dodecyl sulfate-polyacrylamide gel electrophoresis
EMCCD: Electron-multiplying charge-coupled device
