# Supplementary material for: γ-2 and GSG1L bind with comparable affinities to the tetrameric GluA1 core
Source: Cell Mol Biol Lett. 2023 Jul 10;28:54. doi: 10.1186/s11658-023-00470-9 (PMC10332039; doi:10.1186/s11658-023-00470-9)
Supplement: Supplementary file 1 — Additional file 1. Note 1. Rationale for choosing intracellularly injected nanobodies for labeling. Note 2. Model for binding of γ-2 and GSG1L to GluA1. Note 3. Full nanobody sequence. Figure S1. GFP-labeled GSG1L is functional. [file 11658_2023_470_MOESM1_ESM.pdf]

γ-2 and GSG1L bind with comparable affinities to the tetrameric GluA1 core

Chenlu Yu, Hendrik F. P. Runge, Antara Mukhopadhyay, Gerd Zolles, Maximilian H. Ulbrich

Additional File 1

Note 1: Rationale for choosing intracellularly injected nanobodies for labeling

Note 2: Model for binding of γ-2 and GSG1L to GluA1

Note 3: Full nanobody sequence

Figure S1: GFP-labeled GSG1L is functional

Note 1: Rationale for choosing intracellularly injected nanobodies for labeling

For three-color single-molecule imaging, we needed three spectrally separated tags. Since GFP and mCherry cover the green and orange-red emission range, an additional color can be placed either in the blue or in the far-red range. Because the Olympus 100x NA 1.70 objective, which we use due to its high signal-to-noise ratio for single-molecule imaging, requires an immersion liquid that does not transmit wavelengths below 480 nm well, we could not use blue or cyan fluorescent proteins. There are no sufficiently bright fluorescent proteins in the far-red range that can be spectrally separated from mCherry, and therefore we resorted to using an organic dye.

One possibility would be the use of a tagging system like the SNAP-tag where an extracellular tag at the target protein can be labeled by a fluorescent substrate that is supplied through the medium. However, the *Xenopus* oocytes are covered by the vitelline membrane, a gel-like layer where the externally applied substrate gets entangled. Although the vitelline membrane is being removed immediately before placing the cell on the coverslip for imaging, it is impossible to effectively wash the cell because without the vitelline membrane, it is delicate and ruptures easily. On the other hand, for the planned experiments it is also not possible to resort to a different cellular expression system where extracellular labeling works well (e.g. mammalian cell lines). The reason is that in *Xenopus* oocytes, ionotropic glutamate receptors and some other membrane proteins remain immobile in the membrane (for unknown reasons), and therefore facilitate counting photobleaching steps of attached fluorescent proteins. In mammalian cells, these proteins are mobile, which makes it impossible to reliably count photobleaching steps. In addition, *Xenopus* oocytes allow the control of expression levels by adjusting the amount of injected RNA.

We therefore came up with the idea to use an epitope tagging system intracellularly and inject the substrate into the cell. As it is impossible to wash out the substrate after labeling, once it is injected into the oocyte, we searched for a tagging system that requires only a low concentration of substrate to minimize the background, and chose the anti-GFP nanobody, which binds to GFP with an affinity below 1 nM and has previously been used for single-molecule imaging of membrane proteins in mammalian cells [13−15].

A647-Nb contained a total of 4 solvent-exposed lysines that could potentially have been labeled with dye NHS esters, and had an average of 1.0−1.5 A647 dye labels attached; assuming all solvent-exposed lysines have an equal chance of getting labeled, the number of labels per A647-Nb molecule should be binomially distributed. Accordingly, determining the subunit number of the target protein by counting the photobleaching steps from the dye is difficult.

Note 2: Model for binding of γ-2 and GSG1L to GluA1

To model the binding of γ-2 and GSG1L to GluA1, we assume that the densities are in an equilibrium for all assemblies. From Fig. 5A we see:

$$A + G \underset{k_{Goff}}{\overset{4\,k_{Gon}}{\rightleftharpoons}} AG1$$

$$AG1 + G \underset{2\,k_{Goff}}{\overset{3\,k_{Gon}}{\rightleftharpoons}} AG2$$

etc. Then

$$[AG1] = 4\,K_G\,[A][G]$$

$$[AG2] = 3/2\,K_G\,[AG1][G] = 6\,{K_G}^2\,[A][G]^2$$

$$[AG3] = 2/3\,K_G\,[AG2][G] = 4\,{K_G}^3\,[A][G]^3$$

$$[AG4] = 1/4\,K_G\,[AG3][G] = {K_G}^4\,[A][G]^4$$

$$[A\gamma 1] = 4\,K_\gamma\,[A][\gamma]$$

$$[A\gamma 2] = 3/2\,K_\gamma\,[A\gamma 1][\gamma] = 6\,{K_\gamma}^2\,[A][\gamma]^2$$

$$[A\gamma 3] = 2/3\,K_\gamma\,[A\gamma 2][\gamma] = 4\,{K_\gamma}^3\,[A][\gamma]^3$$

$$[A\gamma 4] = 1/4\,K_\gamma\,[A\gamma 3][\gamma] = {K_\gamma}^4\,[A][\gamma]^4$$

$$[AG1\gamma 1] = 3\,K_\gamma\,[AG1][\gamma] = 12\,K_G\,K_\gamma\,[A][G][\gamma]$$

$$[AG1\gamma 2] = K_\gamma\,[AG1\gamma 1][\gamma] = 12\,K_G\,{K_\gamma}^2\,[A][G][\gamma]^2$$

$$[AG1\gamma 3] = 1/3\;K_\gamma\,[AG1\gamma 1][\gamma] = 4\,K_G\,{K_\gamma}^3\,[A][G][\gamma]^3$$

$$[AG2\gamma 1] = 2\,K_\gamma\,[AG2][\gamma] = 12\,{K_G}^2\,K_\gamma\,[A][G]^2[\gamma]$$

$$[AG2\gamma 2] = 1/2\;K_\gamma\,[AG2\gamma 1][\gamma] = 6\,{K_G}^2\,{K_\gamma}^2\,[A][G]^2[\gamma]^2$$

$$[AG3\gamma 1] = K_\gamma\,[AG3][\gamma] = 4\,{K_G}^3\,K_\gamma\,[A][G]^3[\gamma]$$

Because not all fluorescent protein molecules are functional, the bleaching steps counts do not equal the actual numbers of auxiliary subunits. We defined the probability of mNeonGreen to be functional as p and of mCherry as q. Since the receptors contained four GluA1-GFP(Y66L) and were usually labeled with multiple A647-Nb, we approximated that all receptor cores were visible (see Fig. 3C). Therefore, we could write the concentration of counted spots as:

$$[G_{count}] = [G]\,p$$

$$[\gamma_{count}] = [\gamma]\,q$$

$$\begin{aligned}[A_{count}] = [A] &+ [AG1]\,(1-p) + [AG2]\,(1-p)^2 + [AG3]\,(1-p)^3 + [AG4]\cdot(1-p)^4 \\ &+ [A\gamma 1]\,(1-q) + [A\gamma 2]\,(1-q)^2 + [A\gamma 3]\,(1-q)^3 + [A\gamma 4]\,(1-q)^4 \\ &+ [AG1\gamma 1]\,(1-p)\,(1-q) + [AG2\gamma 1]\,(1-p)^2\,(1-q) \quad + [AG3\gamma 1]\,(1-p)^3\,(1-q) \\ &+ [AG1\gamma 2]\,(1-p)\,(1-q)^2 + [AG2\gamma 2]\,(1-p)^2\,(1-q)^2 + [AG1\gamma 3]\,(1-p)\,(1-q)^3\end{aligned}$$

$$[AG1_{count}] = [AG1]\, p + 2\, [AG2]\, p\, (1-p) + 3\, [AG3]\, p\, (1-p)^2 + 4\, [AG4]\, p\, (1-p)^3 + [AG1\gamma1]\, p\, (1-q) + 2\, [AG2\gamma1]\, p\, (1-p)\, (1-q) + 3\, [AG3\gamma1]\, p\, (1-p)^2\, (1-q) + [AG1\gamma2]\, p\, (1-q)^2 + 2\, [AG2\gamma2]\, p\, (1-p)\, (1-q)^2 + [AG1\gamma3]\, p\, (1-q)^3$$

$$[AG2_{count}] = [AG2]\, p^2 + 3\, [AG3]\, p^2\, (1-p) + 6\, [AG4]\, p^2\, (1-p)^2 + [AG2\gamma1]\, p^2\, (1-q) + 3\, [AG3\gamma1]\, p^2\, (1-p)\, (1-q) + [AG2\gamma2]\, p^2\, (1-q)^2$$

$$[AG3_{count}] = [AG3]\, p^3 + 4\, [AG4]\, p^3\, (1-p) + [AG3\gamma1]\, p^3\, (1-q)$$

$$[AG4_{count}] = [AG4]\, p^4$$

$$[A\gamma_{count}] = [A\gamma1]\, q + [A\gamma2]\, (1-(1-q)^2) + [A\gamma3]\, (1-(1-q)^3) + [A\gamma4]\, (1-(1-q)^4) + [AG1\gamma1]\, (1-p)\, q + [AG2\gamma1]\, (1-p)^2\, q + [AG3\gamma1]\, (1-p)^3\, q + [AG1\gamma2]\, (1-p)\, (1-(1-q)^2) + [AG2\gamma2]\, (1-p)^2\, (1-(1-q)^2) + [AG1\gamma3]\, (1-p)\, (1-(1-q)^3)$$

$$[AG1\gamma_{count}] = [AG1\gamma1]\, p\, q + 2\, [AG2\gamma1]\, p\, (1-p)\, q + 3[AG3\gamma1]\, p\, (1-p)^2\, q + [AG1\gamma2]\, p\, (1-(1-q)^2) + 2\, [AG2\gamma2]\, p\, (1-p)\, (1-(1-q)^2) + [AG1\gamma3]\, p\, (1-(1-q)^3)$$

$$[AG2\gamma_{count}] = [AG2\gamma1]\, p^2\, q + 3\, [AG3\gamma1]\, p^2\, (1-p)\, q + [AG2\gamma2]\, p^2\, (1-(1-q)^2)$$

$$[AG3\gamma_{count}] = [AG3\gamma1]\, p^3\, q$$

Because we could not distinguish 1, 2, 3 or 4 subunits of γ-2-mCherry, we used:

$$[A\gamma_{count}] = [A\gamma1_{count}] + [A\gamma2_{count}] + [A\gamma3_{count}] + [A\gamma4_{count}]$$

$$[AG1\gamma_{count}] = [AG1\gamma1_{count}] + [AG1\gamma2_{count}] + [AG1\gamma3_{count}]$$

$$[AG2\gamma_{count}] = [AG2\gamma1_{count}] + [AG2\gamma2_{count}]$$

$$[AG3\gamma_{count}] = [AG3\gamma1_{count}]$$

With the above equations and the counted number of protein complexes, we could fit the K_G and K_γ by least-square fitting. In practice, p was set to 0.77, which was fitted from GluA1-mNeonGreen experiments, and q was set to 0.65, which was our estimate from previous results and was also determined independently [17].

Note 3: Full nanobody sequence

MKYLLPTAAAGLLLLAAQPAMAMDKSKSGKSGKGSDPQVQLVESGGALVQPGGSLRLSCAASGF
PVNRYSMRWYRQAPGKEREWVAGMSSAGDRSSYEDSVKGRFTISRDDARNTVYLQMNSLKPE
DTAVYYCNVNVGFEYWGQGTQVTVSSKLAAALEHHHHHH

Green: PelB leader sequence

Orange: 4x lysine tag for labeling

Blue: anti-GFP nanobody

Red: linker and 6x His tag

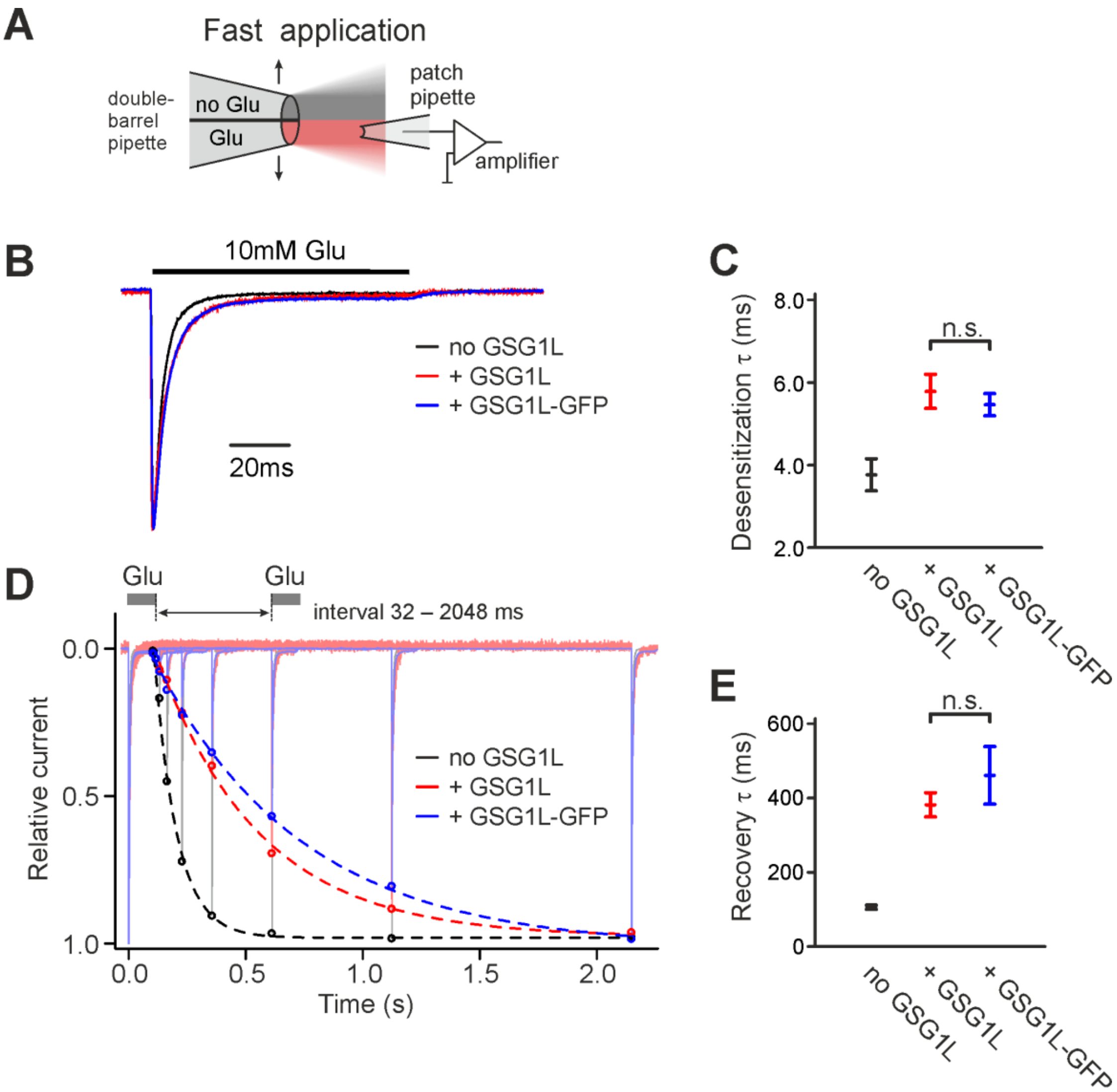

Figure S1: GFP-labeled GSG1L is functional. (A) Current responses from outside-out patches were elicited by rapid application/removal of glutamate using a piezo-controlled fast application system with a double-barrel application pipette. (B) Representative current responses of AMPARs recorded upon 100 ms applications of 10 mM glutamate (black bar above current traces) from GluA1+GluA2 alone (black) or in combination with either GSG1L (red) or GSG1L-GFP (blue). (C) Time constants of desensitization for GluA1+GluA2 alone or with either GSG1L or GSG1L-GFP (n=4−8; mean±s.e.m.). GSG1L-GFP slows down the desensitization like untagged GSG1L. (D) Recovery of AMPARs with composition as in (B) from steady-state desensitization recorded with a double-pulse protocol (pair of two 100 ms glutamate pulse separated by increasing time intervals). Circles indicate peak currents recorded during the second pulse. Dashed lines are mono-exponential fits to the time course of the peak currents. (E) Recovery time constants (n=4−8; mean±s.e.m.). GSG1L-GFP slows down the recovery from desensitization like untagged GSG1L.
